# Fresh and strength properties of high volume ultra-fine fly ash cement mortar with calcinated limestone powder

**DOI:** 10.1038/s41598-025-33895-7

**Published:** 2025-12-27

**Authors:** J. Rajesh, S. Kandasamy, Ashish Agrawal, S. P. Samal, G. Swaminathan

**Affiliations:** 1 Department of Civil Engineering, Vel Tech Rangarajan Dr. Sagunthala R&D Institute of Science and Technology, Chennai, 600062 Tamil Nadu India; 2 Department of Mechanical and Industrial Engineering, Manipal Institute of Technology, Manipal Academy of Higher Education, Manipal, India; 3 Department of Biosciences, Saveetha School of Engineering, Saveetha Institute of Medical and Technical Sciences, Chennai, 602105 India; 4 Department of Civil Engineering, National Institute of Technology, Tiruchirappalli, 620015 Tamil Nadu India

**Keywords:** Low carbon composite cement, Composite cementitious binder, Calcinated lime powder, Bauxite powder, High volume UFFA mortar, Engineering, Environmental sciences, Materials science

## Abstract

The growing demand for sustainable building materials has led to an increased quest for substitute cementitious systems that can mitigate environmental impact while maintaining the characteristics of traditional cementitious systems. Ordinary Portland cement (OPC) production contributes significantly to CO₂ emissions, necessitating the development of sustainable alternatives. Fly ash, being an industrial by-product, is used with cement to make high-volume fly ash blended (HVFA) cement, which offers substantial environmental and economic benefits by limiting the use of ordinary Portland cement. Yet, its extensive usage is hindered by performance downsides such as slower early-age strength and deferred setting time. This study aims to develop a sustainable mortar system by integrating multiple industrial by-products, including ultra-fine fly ash (UFFA), calcinated limestone powder (CLP), and bauxite powder (BP), activated with alkaline activators (AA). The primary objective is to replace 40–60% of ordinary Portland cement, thereby improving the mechanical and durability properties of the mortar compared to conventional mortar systems. In the production of sustainable mortar, incorporating 40–60% of ultra-fine fly ash, 10–20% of calcinated limestone powder, 0–10% of bauxite powder, and 3–9 g of alkaline activator. Mortar specimens were prepared with varying proportions of industrial by-products: 40–60% ultra-fine fly ash, 10–20% calcinated limestone powder, 0–10% bauxite powder, and 3–9 g of alkaline activator, maintaining a 1:3 binder-to-fine aggregate ratio. Comprehensive testing was implemented, including workability assessment, setting time determination, compressive strength evaluation, flexural strength testing, fracture toughness analysis, and detailed microstructural characterisation. The optimum mixture composition, consisting of 40% ordinary Portland cement, 40% ultra-fine fly ash, 15% low thermal calcinated lime powder, and 5% bauxite powder with alkaline activator made from 6 g of magnesium carbonate powder, demonstrated superior performance characteristics. This optimal blend achieved enhanced mechanical properties compared to conventional mortar while successfully replacing 60% of ordinary Portland cement with industrial by-products. The improved performance of the optimum mixture can be attributed to synergistic effects between ultra-fine fly ash, calcinated limestone powder, and bauxite powder under alkaline activation. Future research should investigate the long-term durability performance of the developed sustainable mortar under various environmental exposure conditions.

## Introduction

As a well-known fact of adding fly ash (FA) into the cement as a binder is demonstrated over decades in cement manufacturing, in this process the quantity of fly ash amalgamation was restricted to a maximum of 33 to 35% to make an ordinary Portland cement (OPC) to pozzolanic Portland cement (PPC) as per IS: 3812: Part 1: 2013^1,2^ Meanwhile, growing demand for low-cost, sustainable, and eco-friendly cement needs multiple research on identifying various supplementary cementitious materials which can be combined with nominal cement, thereby reducing the quantity of cement consumption^3–7^.

The construction industry faces mounting pressure to reduce its environmental footprint, particularly regarding cement production, which accounts for approximately 8% of global CO₂ emissions. Traditional Portland cement production is energy-intensive and contributes significantly to greenhouse gas emissions, necessitating the development of sustainable alternatives^8–11^. This study focuses on utilising industrial by-products, which possess pozzolanic characteristics, such as fly ash, limestone quarry waste powder, and bauxite powder, as a partial replacement of OPC. By keeping the fly ash as a major replacement element (40–60%), thereby ending up with a high-volume fly ash cement mix, which is eco-friendly and sustainable. Nevertheless, this cement mix has demerits, including a lack of early strength development, prolonged setting time, and leaching of concrete over time.

Hence, to mitigate the demerits of high-volume fly ash^12,13^, fly ash was made into ultrafine fly ash (UFFA), and calcination of limestone waste at low temperature (400° C to 450° C) is done to form calcinated lime powder (CLP)^14,15^ And lumped bauxite residue was made into fine powder to form bauxite powder (BP)^16^. These components were amalgamated in certain proportions and mixed with OPC. UFFA, CLP, and BP were incorporated to counteract the deficiency of silica, calcium and alumina while employing high volume fly ash.

The binding ability of fly ash is profoundly dependent on the type of fly ash used, such as class C or class F, as the constituents of class F and class C are different^17^. Adding class F-type fly ash leads to the deprivation of calcium, aluminium, and their associated oxides. When the calcium deficits occur, the reactivity of fly ash-based cement with water breaches, resulting in a fragmentary chemical reaction which forms incomplete or less of Bogue’s components^18^. This will be contradicted by adding calcinated limestone powder along with bauxite powder to counter calcium and alumina deficiency^19–22^. The alkalinity of alkaline activator stimulates the hydration process and the formation of Alumino-Ferrite-tetrahydate (AFt)^23^ phase with incorporating a diminutive amount of bauxite powder^24^.

The bauxite residue is an industrial waste by-product generated during aluminium production using the Bayer process^25^. In 2024, India consumed around 4.5 million metric tons of aluminium, and it is predicted to be 6 to 6.5 million metric tonnes by the end of 2025^26^. 1.5 to 2 tons of bauxite residue are generated as byproduct for every tonne of aluminium produced, hence the utilisation of bauxite powder (BP) into concrete^27^. It can become one of the safe ways of disposal of bauxite residue.

India produced around 450 million metric tonnes of limestone in 2024^28^, approximately 15% of the total limestone produced becomes waste constituent by material handling and processing techniques. Hence, roughly 67.5 million metric tonnes of limestone quarry powder were produced in India. The lime powder waste is collected from Yerraguntla industrial village, where the powder is abundantly available, located in Andhra Pradesh, India. Incorporating limestone quarry powder into mortar makes sense to reduce the usage of raw limestone in cement production. Calcination of limestone debris will be done with the combustion synthesis technique^29^ to reduce the rate of temperature for calcination from 900 °C to 450 °C using a muffle furnace. As a result of calcination, calcium hydroxide is readily formed, which further reacts with other elements during hydration and forms Calcium-silicate-hydrate (C-S-H) components^30^.

Preliminary treatments are done for all waste materials to make them ready for bonding, regarding ultra-fine fly ash, calcinated lime powder and bauxite powder. Drying, screening, grinding, and sieving were done. Fly ash is made into ultrafine fly ash by grinding in a ball mill. Calcination is done for limestone quarry debris and powder^31^. Regarding the Alkaline activator, removal of impurities, neutralisation of pH^32^ and physical stabilisation were performed. The bauxite powder has a high pH due to densely concentrated sodium hydroxide and calcium along with composite composition of chemicals^33^ which makes the bauxite has a solid waste. The chemical composition is cementitious in nature hence, slightly modifying by grinding makes it has a perfect cementitious supplementary.

Nevertheless, due to high content of silica in fly ash makes the fly ash more reactive with other cementitious elements. The volume of reactive silica in fly ash plays a significant role in quantifying the volume of fly ash that can be infused with cement as a blender for mortar and concrete. Previous studies primarily investigated individual waste materials (fly ash, limestone, or bauxite powder) or binary combinations, with minimal research on ternary waste systems.

While various industrial by-products have been explored as partial cement replacements, the simultaneous utilization of multiple waste streams-specifically the combination of Ultrafine Fly Ash, Calcined Limestone Powder, and bauxite Powder in alkaline-activated systems remains underexplored. While alkaline activation of individual waste materials has been studied, the synergistic effects of combining ultra-fine fly ash, lime powder, and bauxite residue under higher limit alkaline conditions remain poorly understood. Most existing studies focus on single or dual waste material combinations, limiting the potential for maximizing waste utilization and optimizing concrete properties. Hence, this experimental study proposes to examine the impacts on combined effect of the ternary mix on engineering parameters like workability, setting time, strength, and microstructural behaviour of mortar specimens.

## Constituent materials

Ordinary Portland cement used is of 53 grade complies with IS: 12,269 − 2013^34^ with specific gravity as per IS: 4031: Part 11: 2024^35^ as 3.14 and 246 m^2^/kg as specific surface area determined as per IS: 4031: Part 1: 2021^36^. Natural river sand is used as fine aggregate with Zone II grading with fineness modulus of 2.96 complying to IS: 383–2021^37^. The fine aggregate is quarried from Arani River of Tiruvallur district, Tamil Nadu. This sand falls under medium sized particle size ranging from 4.75 mm to 0.15 mm. The water absorption of fine aggregate was 1.76% complying with IS: 2386: Part 3: 2021.

**Fig. 1:**
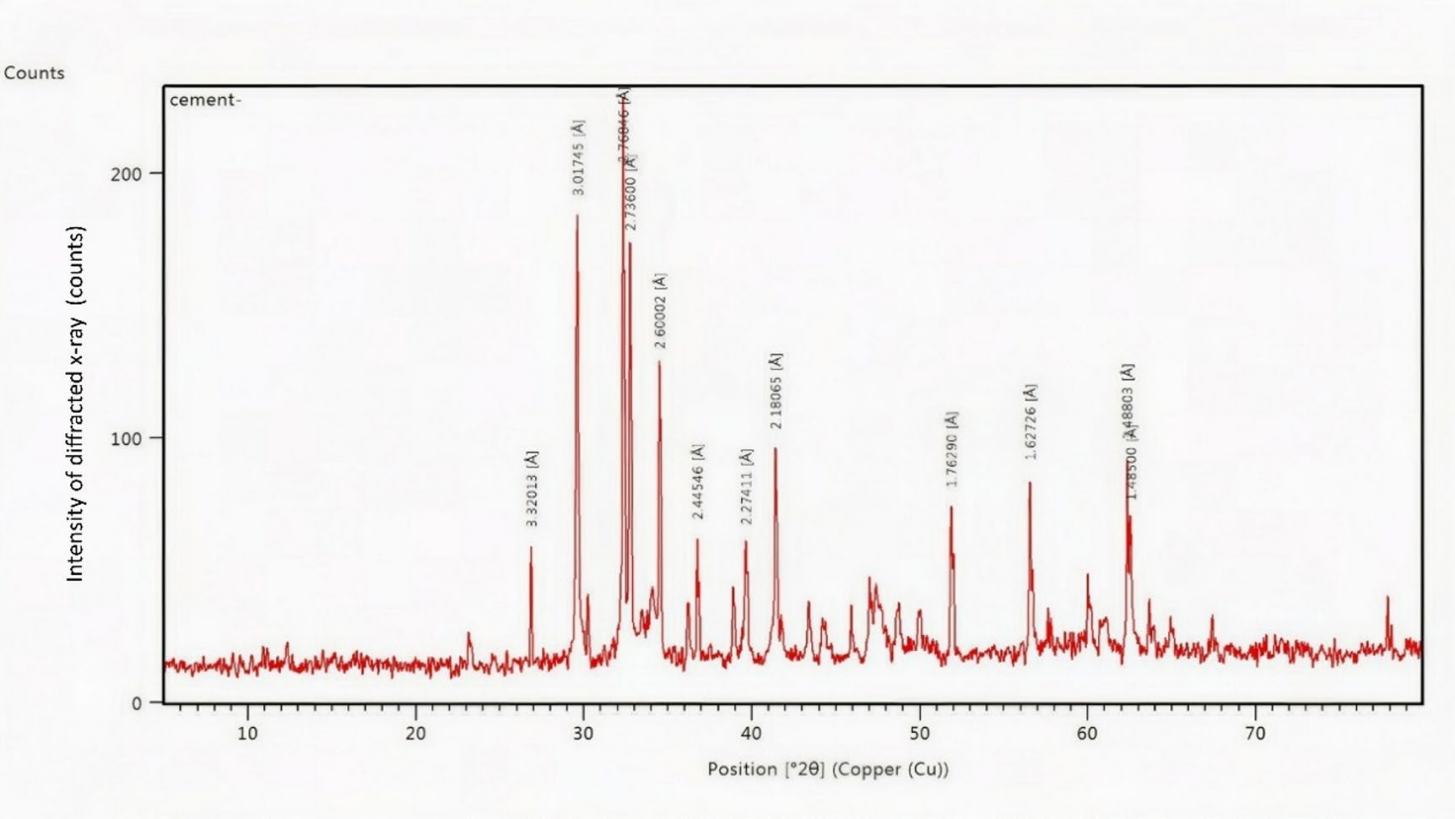
X-ray Diffraction analysis of cement.

**Fig. 2:**
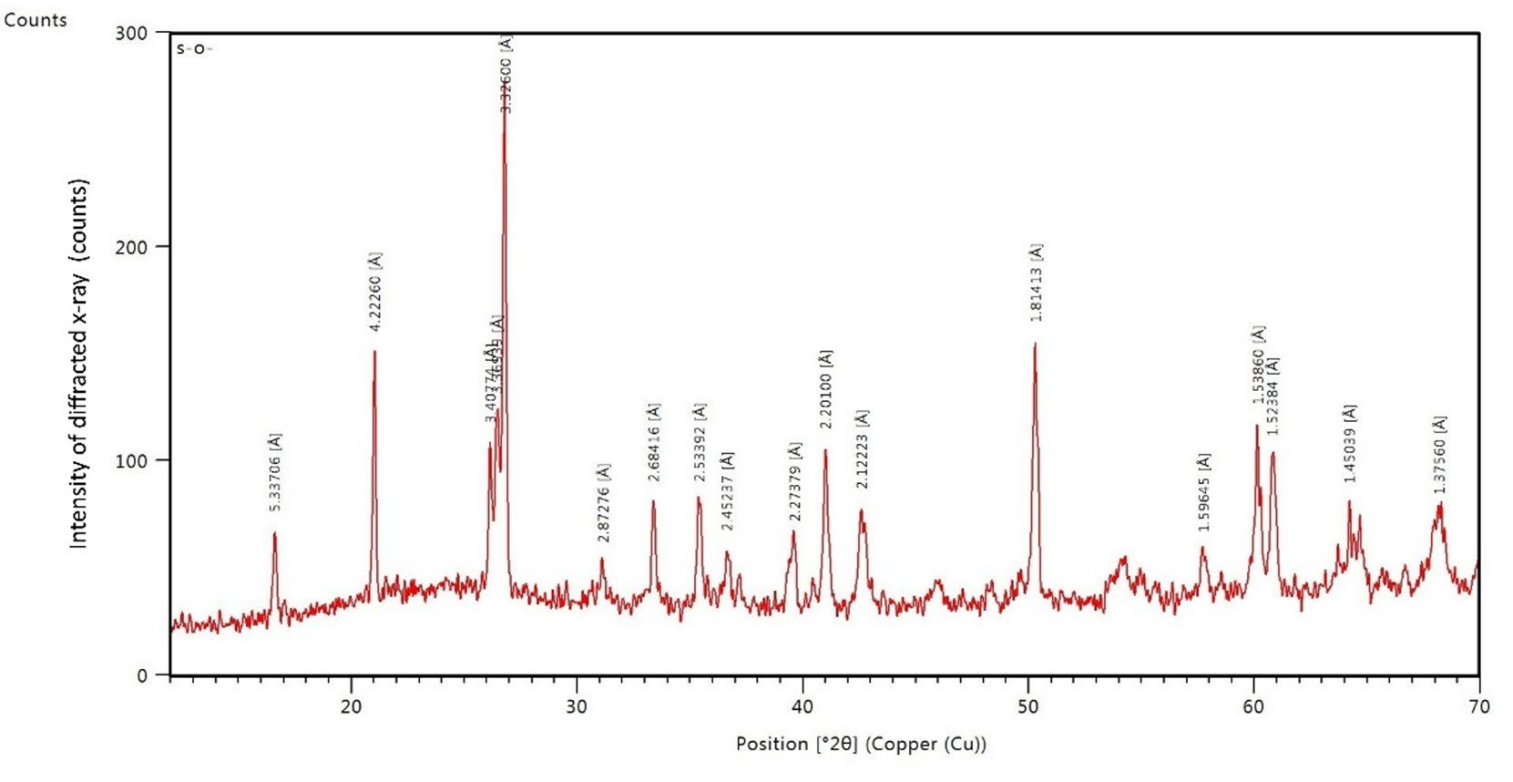
X-ray Diffraction analysis of ultra-fine fly ash.

X-ray fluorescence analysis results which are listed in Table 1 and illustrated in Figs. 1 and 2 were done by National Centre for Earth Science Studies, Kerala. The chemical constituent of ordinary Portland cement, ultra-fine fly ash, calcinated limestone powder and red mud also known as bauxite powder were tabulated Table 1 by percentile in mass. Fly ash of class F with particle size D_90_ as 5 to 7 microns with loss of ignition as 2.64, complying with IS: 3812: Part 1: 2013^2^ is adopted. Fly ash was taken from Ennore thermal power station dump yard, Tamil Nadu, India.

Bauxite residue being a byproduct of aluminium production which is highly alkaline, caustic and harmful in nature requires neutralization before reuse. To neutralize bauxite residue, the most effective and economic approach adopted was treating with gypsum which contains Mg^2+^ and Ca^2+^ that reacts with hydroxide to neutralize the pH. The lump of bauxite residue is ground in ball mill to the fineness of cement and mixed with other cementitious matrix to form hybrid high volume ultra-fine fly ash mortar.

**Table 1:** Chemical components of OPC, UFFA, CLP and BP.

Elements	SiO_2_	Al_2_O_3_	Fe_2_O_3_	TiO_3_	MnO	CaO	MgO	K_2_O	SO_3_	Na_2_O	*P*_2_O_5_
OPC (%)	21.9	6.5	3.47	-	0.08	60.8	0.95	0.6	3.31	1.21	0.23
UFFA(%)	58.3	27.5	5.05	2.22	0.03	1.85	0.53	1.59	0.11	0.08	0.47
CLP (%)	6.4	2.7	1.15	-	-	81.7	6.05	-	-	1.2	-
BP (%)	11.6	16.54	52.3	-	0.04	8.33	3.3	-	0.02	4.65	-

Chemical constitutes listed in Table 1 shows the ability of the treated industrial wastes to become supplementary cementitious material while mixing with OPC. In Figs. 1 and 2, the ‘y’ axis sharp peaks show the intensity of diffracted x rays of highly crystalline and broad peaks shows amorphous elements of which are ready for reaction during hydration^35^. The major peaks position of (2θ°) was observed between 12° to 65°.

Figure 2 represents the X-ray diffraction analysis of ultra-fine fly ash; the fly ash used for this study complies with IS: 3812: Part 1: 2013^2^. The plot illustrates the multiple sharp peaks corresponding for different chemical elements of crystalline form meanwhile broad peaks are also available which emphases the presence of amorphous state^39^ which are the key elements in binding of FA with cement particles^40^. The major peaks position of (2θ)° was observed between 15° to 70° which was broader than cement.

The Polycarboxylate Ether (PCE) is used as superplasticizers (SP) to improvise the workability and confined packing of cement particles to achieve expected strength with low water to cement ratio of 0.3^41^. Master Glenium SKY 8233 superplasticizer is used in this research, which is an industrial standard product. This superplasticizer acts as a repelling agent between individual cement particles for a period i.e., from initial setting time to final setting time. The repelling strength diminishes as time elapses from initial setting time to final setting time. Tap water complying with IS: 456–2024^38^ is used in this study. The required dosage of PCE is determined by the target characteristic compressive strength, the water-to-cementitious-powder (w/cp) ratio, and the cement type. As the w/cp ratio decreases, the superplasticizer demand increases. Typically, the dosage ranges from 500 ml to 1500 ml per 100 kg of cementitious materials, with a maximum usage limit of 1.0% by weight of the cementitious content.

## Preparation of materials

Being a quaternary matrix (OPC, UFFA, CLP, BP) with ternary industrial waste materials, the term “preparing the materials” focusing on making the materials to be reactive while blended with OPC. Ultra-fine Fly ash is prepared by grinding the fly ash, for D_90_ the size of particles should be 5 to 7 microns^42^ which is ensured by grinding in ball mill. limestone quarry debris was calcinating at 450° Celsius using muffle furnace [29] while mixed charcoal. Sieving is done to separate the charcoal and lime powder meanwhile ensuring uniform well graded particle size, treating with thermal process of around 400 °C to 450 °C in muffle furnace weakens the bond between carbon and oxygen which makes calcium carbonate more reactive to form calcium oxide which reacts with other ingredients.

Bauxite residue is grinded in tumbler type ball mill till cement like particle size is achieved. BP will promote magnesium–aluminium–iron compound (Mg-Al-(Fe)) in amorphous phase^43^, by merging with fly ash and lime powder which is one of the key components for later strength development. The alkaline activator (AA) used in this work was prepared by taking 3 g, 6 g, and 9 g of magnesium carbonate powder per one litre of water and soaked for 24 h. After sedimentation, the clear water on the top is decanted into a glass beaker which is used as alkaline activator. The AA which shows pH less than 12 and more than 13 will not be considered for examining, hence the range of 3 g to 9 g is adopted.

### Mix proportions

For preparing mortar, cementitious powders were proportionated as listed in Table 2. The ratio of binder to fine aggregate is kept as 1:3 i.e., 590 kg of cement/binder and 1770 kg of fine aggregate for 1m^3^ of mortar. In binder constituent, a minimum limit of cement is kept as 30% and in remaining 70%, 50% will be ultra-fine fly ash and the lasting proportion will be calcinated lime powder and bauxite powder. Maximum limit of ternary mix was kept as 50%, 20% and 10% respectively. Natural sand with zone II is used as fine aggregate for all mixes along with 1% of SP.

Table 2 also shows the mix proportions and quantities of all the quaternary components mix measured for 1 L of mortar with 10% of contingency. Cube mortar of 70.6 mm^3^ complies with IS: 4031: Part 6: 2019^44^ were employed for casting cubes. Dry mixing of the ternary mix is done to ensure proper amalgamation of each element. Proper mixing of binder with water to form mortar is ensured by using electrical mortar mixer of 6 L capacity complies with IS: 2250–2020^45^.

**Table 2:** Mixture proportion of binding materials for mortar.

S. no	Mix ID	Mix description	OPC	FA	UFFA	CLP	BP
			(grams)				
1	CS	Control specimen	590	-	-	-	-
2	A2	C40:F60:L0:B0	236	354	-	-	-
3	A3	C40:UF60:L0:B0	236	-	354	-	-
4	B1	C30:UF50:L10:B10	177	-	295	59	59
5	B2	C30:UF50:L15:B5	177	-	295	88.5	29.5
6	B3	C30:UF50:L20:B0	177	-	295	118	-
7	C1	C35:UF45:L10:B10	206.5	-	265.5	59	59
8	C2	C35:UF45:L15:B5	206.5	-	265.5	88.5	29.5
9	C3	C35:UF45:L20:B0	206.5	-	265.5	118	-
10	D1	C40:UF40:L10:B10	236	-	236	59	59
11	D2	C40:UF40:L15:B5	236	-	236	88.5	29.5
12	D3	C40:UF40:L20:B0	236	-	236	118	-

*C- OPC, F- Fly ash, UF- UFFA, L- CLP, B- BP: proportion in ‘%’.

## Experimental programmes

### Consistency and setting time

As per IS: 4031: Part 4 and Part 5: 2024^46,47^, the normal consistency and initial and final setting is carried out for all mixes and results were listed in Table 2. The Vicat’s plunger penetration for reading was kept 5–6 mm for bottom of the paste. The mortar is prepared as per the guidelines of IS: 1727–2013^48^ using mortar making machine. The minimum quantity of water required for making the cementitious powder into paste by hydration process with minimum C-S-H gel resulting in forming a semisolid paste which is termed as standard consistency, which represents the ability of cementitious powder to be converted into semi liquid paste by adding water. The semisolid paste form of mortar which as the stiffness slightly more than Bingham type of plastic phase of a fluid is kept for consistency measure. For Initial and final setting time, the quantity of water will be 85% of water required for standard consistency.

### Workability of mortar

The ease of working with given mortar in term of mixing, placing, and compacting of mortar while confirming the adhesion and cohesion properties is established by workability also known as rheology of mortar. Testing was done using a mortar flow table complying with IS: 4031: Part 7: 2019^49^. The quantity of water required for each mix is adopted from the individual consistency results of each mix. The flow table to measure workability of mortar complying with IS: 5512 − 2013^50^ is selected for testing. 25 number of rotations or drops is applied within 15 s, and the spread mortar is measured for its longest possible diameter. The ratio of change in the diameter to the original diameter of the mortar indicates the workability of the specimen.

### Compressive strength

As per IS: 4031 Part 6: 2019^44^, Mortar cubes of 70.6 mm³ were cast for all mixes for compressive strength testing. Ambient Curing is done for 28, 56 and 90 days under room temperature^44^. The optimisation of the Alkaline activator is done by testing the mortar in a hardened state for compression and flexural strength. Cubes were cast using the Alkaline activator, which act as a reactant between cementitious powder elements.

Table 3 shows the density measure of all mortar cubes which confirms that the weight of mortar cubes is with in permissible limit as per IS 2250: 2000, with an average of 770 g per cube. The M1 mix showed the maximum density of 2238 kg/m^3^, and M2 shows the least density of 2096 kg/m^3^, hybrid high volume ultra-fine fly ash mix M11 shows 2228 kg/m^3^.

**Table 3:** Average weight of mortar cubes after curing.

S. no	Mix ID	≈ Avg. weight of 3 Specimens (g)
1	M1	790
2	M2	740
3	M3	753
4	M4	743
5	M5	747
6	M6	756
7	M7	768
8	M8	774
9	M9	780
10	M10	783
11	M11	786
12	M12	786

Curing was done in lime-saturated water to reduce the leaching of calcium oxide from the specimens to prevent the softening of the surface of the specimens. After completion of curing, samples were dried for 8 to 12 h in room conditions before subjecting to testing. Mortar cubes are tested in compressive testing machine at the rate of 35 N/mm^2^. Total of 302 mortar and prism cubes were cast.

### Flexural strength of mortar

**Fig. 3:**
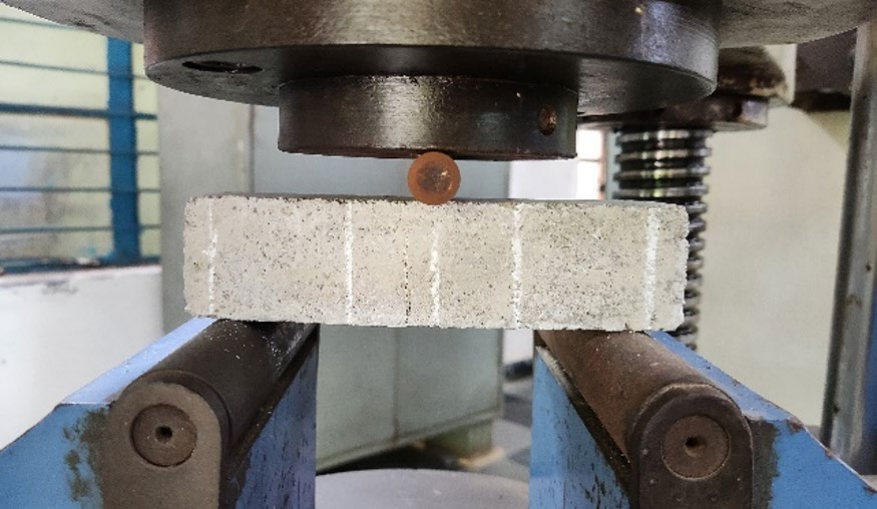
Flexural test setup using UTM.

As per IS: 4031 (Part 6)^44^ The Mortar prism was cast and cured at an ambient temperature of 27℃ ± 2℃. The flexural strength of blended cementitious mortar is observed for the three-point loading test, as shown in Fig. 3. Mortar prism cuboids of 160 mm x 40 mm x 40 mm were cast; in total, 102 prism cuboids were cast. The prisms were tested in universal testing machine (UTM) at the rate of 0.29 kN/min, which is calculated by the interpolation method based on values from IS: 516: Part 1/Sect. 1: 2021^51^. Mortar prisms were cast using the prepared Alkaline activator.

To observe the crack propagation and to determine the stress intensity factor, the rate of loading is reduced up to 60% from the original value. Initially, the loading is increased at a rate of 0.35 N/mm^2^ per minute, i.e., 50% of the original loading rate and kept constant throughout the application of load until the failure of the specimens. UTM with 40 kN capacity is used to do the experiment with a least count of 0.08 kN.

The spacing between supports was kept as 120 mm, and point load applied at the centre of the prism via a notch root radius of 16 mm through a mild steel rod. Three-point loading experiment is preferred over the four-point loading because of the high sensitivity response to minimal load induced, as the mortar prism will show noticeable cracks for a minimum load. Therefore, three-point loading is the most preferred way of testing the flexural strength of a mortar prism.

**Fig. 4:**
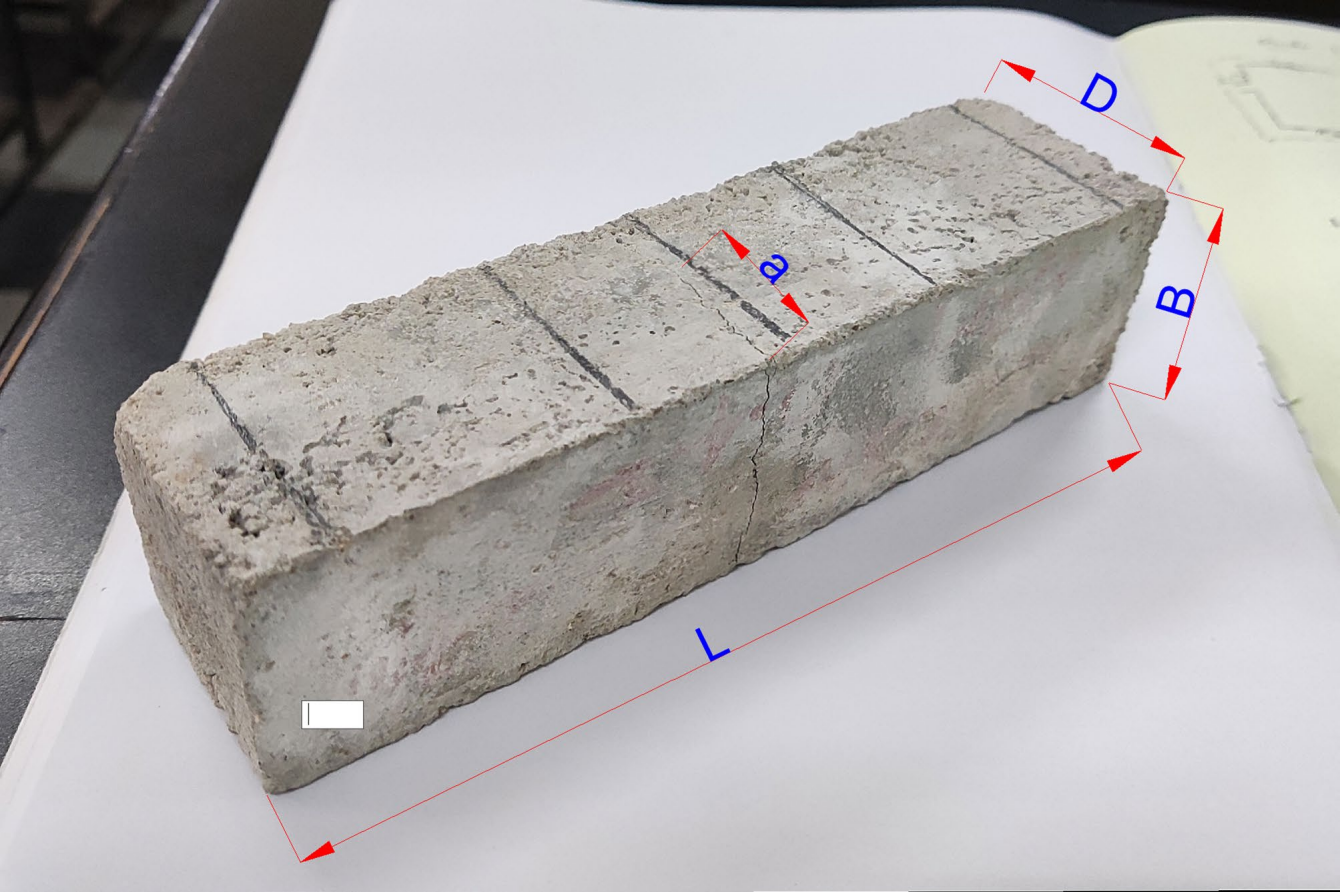
Mortar prism with crack ingress after flexural testing.

To determine the flexural strength (σ_b_) and flexure toughness, also known as Stress intensity factor (K_Ic_) of the isotropic mortar specimens, by marking the depth of crack from the soffit of the prism to its compressive face as shown in Fig. 4, the Figure infers the length of crack development initiated from the tension face. This flexural crack length ‘a is then used to evaluate the stress intensity factor (K_I_) using the following equation, as the prism behaves as a single-edge notch bending specimen. Equation (1) expresses the stress intensity factor of the prism.

1 $$\:{K}_{I}=\frac{3PL}{2B{D}^{3/2}}\left(1.93{\left(\frac{a}{D}\right)}^{1/2}-3.07{\left(\frac{a}{D}\right)}^{\frac{3}{2}}+14.53{\left(\frac{a}{D}\right)}^{\frac{5}{2}}-25.11{\left(\frac{a}{D}\right)}^{\frac{7}{2}}+25.8{\left(\frac{a}{D}\right)}^{\frac{9}{2}}\right)$$

Where, P—Applied critical load, B- Breadth of prism as 40 mm, D- Depth of prism as 40 mm, a—Propagated crack length (in meters), L_eff_—Supported length, i.e., Effective length spanning between support as 120 mm, L—Overall length of prism as 160 mm. The way of evaluating ‘a’ and ‘D’ can be inferred from Fig. 4.

## Results and discussion

### Consistency

**Fig. 5:**
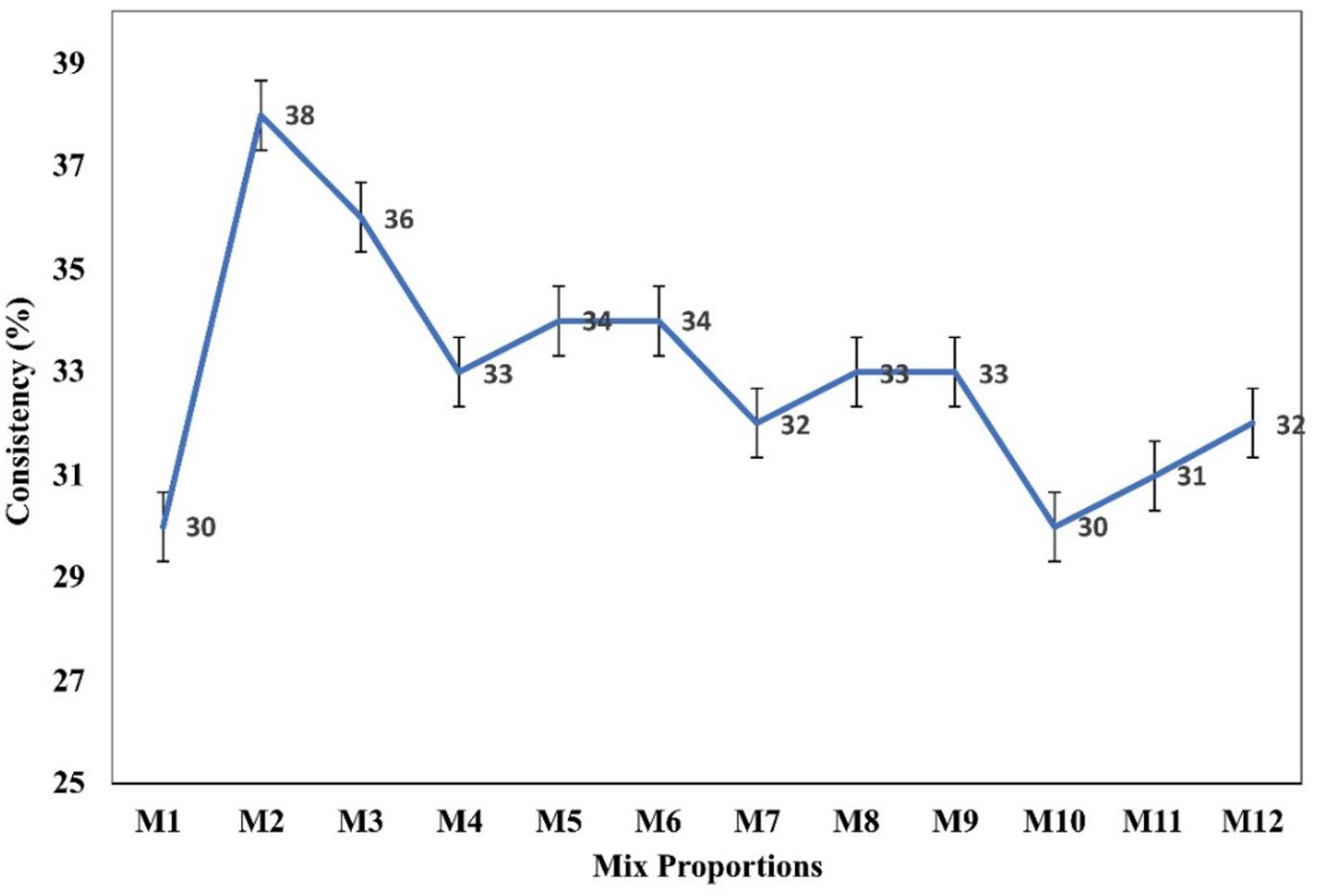
Consistency test results.

The consistency test results were plotted in Fig. 5. The results indicate that M1 and M11 show the highest consistency of 32% followed by M8 and M12 with 31% and M9 with 30%. Consistency plot clearly classifies the blended or hybrid mixes into two groups; (a) mixes with consistency less than 30%, (b) mixes with consistency more than 30%. The high-volume fly ash mix (M2) recorded with least consistency of 20%, due to the very high volume of fly ash with a larger surface area and spherical shape, the demand for water becomes less to form the paste like consistency.

High volume ultra-fine fly ash mix (M3) shows the next least consistency of 24% due to the highest volume of ultra-fine fly ash, which has increased surface area with respect to M2. Hence, water demand is higher than M2. Due to spherical and ultra-fine particle size with smooth surface, ultra-fine fly ash rich mix demands less water to form a paste like state of matter due to ball-bearing effect caused by glassy and even surface of processed fly ash. Therefore, mixes like M3 to M7 show lower consistency. The Blends with and CLP, with almost the same proportion (M8 to M12), show good consistency with a marginal difference in consistency about M1, this is due to the surface attraction and infilling of voids by fine fly ash particles with fine lime particles due to capillary force or electrostatic force while reacting with water. The trial was carried out by 1% increment from the initial iteration.

### Setting time

#### Initial setting time

**Fig. 6:**
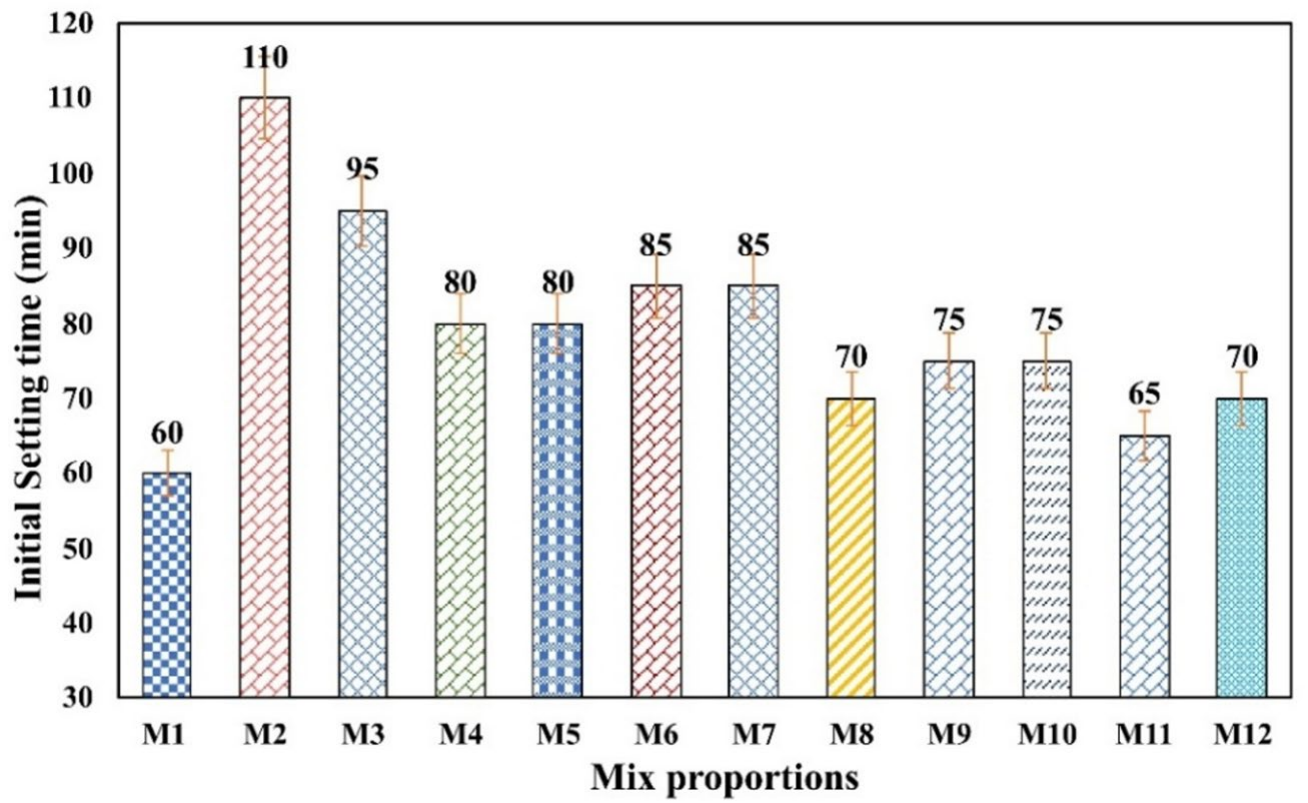
Initial setting time.

The initial setting time test results were plotted in Fig. 6 which indicates the ability of rapid hydration and later hydration. From Fig. 6, it is evident that the control specimen (M1) has a quick setting time, and the high-volume fly ash specimen (M2) shows the longest setting time due to the high volume of fly ash; meanwhile mix M3 attains the setting time within 75% of M2 due to grinded finer particle size. The fine particles, the compacting ability was improved along with the reactivity of fly ash, which leads to a decrease in setting time with respect to M2.

The mix M4 to M7 to M11 shows a higher setting time than M8 to M12 due to the lowest cement content. The mix M11 exhibits almost equal setting time, with just 5 min more than M1. As fly ash is not cementitious in its own nature, when mixed with a smaller amount of clinker as mix M2, the rate of hydration will be slow for two reasons: (a) fly ash itself will react slowly with cement particles due to diluted OPC and exhibit heat during hydration. (b) The exhibited heat will be suppressed by fly ash itself, leading to a low temperature, which can slow down the chemical reaction, making the cycle of hydration further sluggish.

By adding adequate amounts of lime powder as a calcium source, red mud as an alumina source, and ultra-fine fly ash as a reactive silica source, the setting time showed a notable improvement. With varying proportions of these additives (UFFA, CLP and BP), different setting times were achieved. Mix M11, containing an equal amount of OPC and UFFA along with partial inclusion of CLP and a small quantity of BP, exhibited a setting time most comparable to M1. This is likely due to the balanced replacement of OPC with OPC-like constituents, which function as cementitious binders and readily participate in hydration. As a result, the setting time from M8 to M12 decreased significantly.

#### Final setting time

**Fig. 7:**
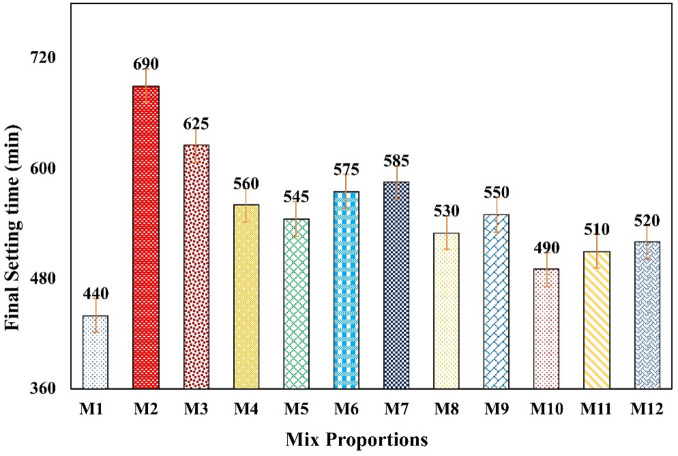
Final setting time.

Figure 7 depicts the final setting time test results; this figure expresses a totally different statement of setting time when compared to the initial setting time. The control mix M1 shows a well-defined setting time as per IS: 12,269 − 2013^34^. Which is closely followed by mix M11. The mix M11 shows 8% more setting time, and mix M8 and M10 show 16% more setting time pertaining to M1. Meanwhile, M2 with a high volume of fly ash show the longest setting time. Due to high pozzolanic nature of fly ash the reactivity with C-H components of cement was sluggish in M2; it took 12 h for complete final setting. The high volume ultra-fine fly ash mix M3 shows a hour reduction in setting time due to ultra-fineness of fly ash. Mix M8 to M12 shows 36 min less setting time on average with respect to mix M4 to M7.

This may be due to the adjusted volume of CLP and BP in Mix 8 to Mix 11 there was a substantial acceleration in pozzolanic reactions, especially between UFFA, calcium hydroxide, and reactive alumina. As the volume of BP is controlled, the alkalinity raises the component’s pH, which further promotes faster activation of the pozzolanic reaction at a later stage of time consumption. Moreover, the duration of final setting time of mix M8 to M11 was within prescribed limit as per IS: 12269:2013.

### Workability

**Fig. 8:**
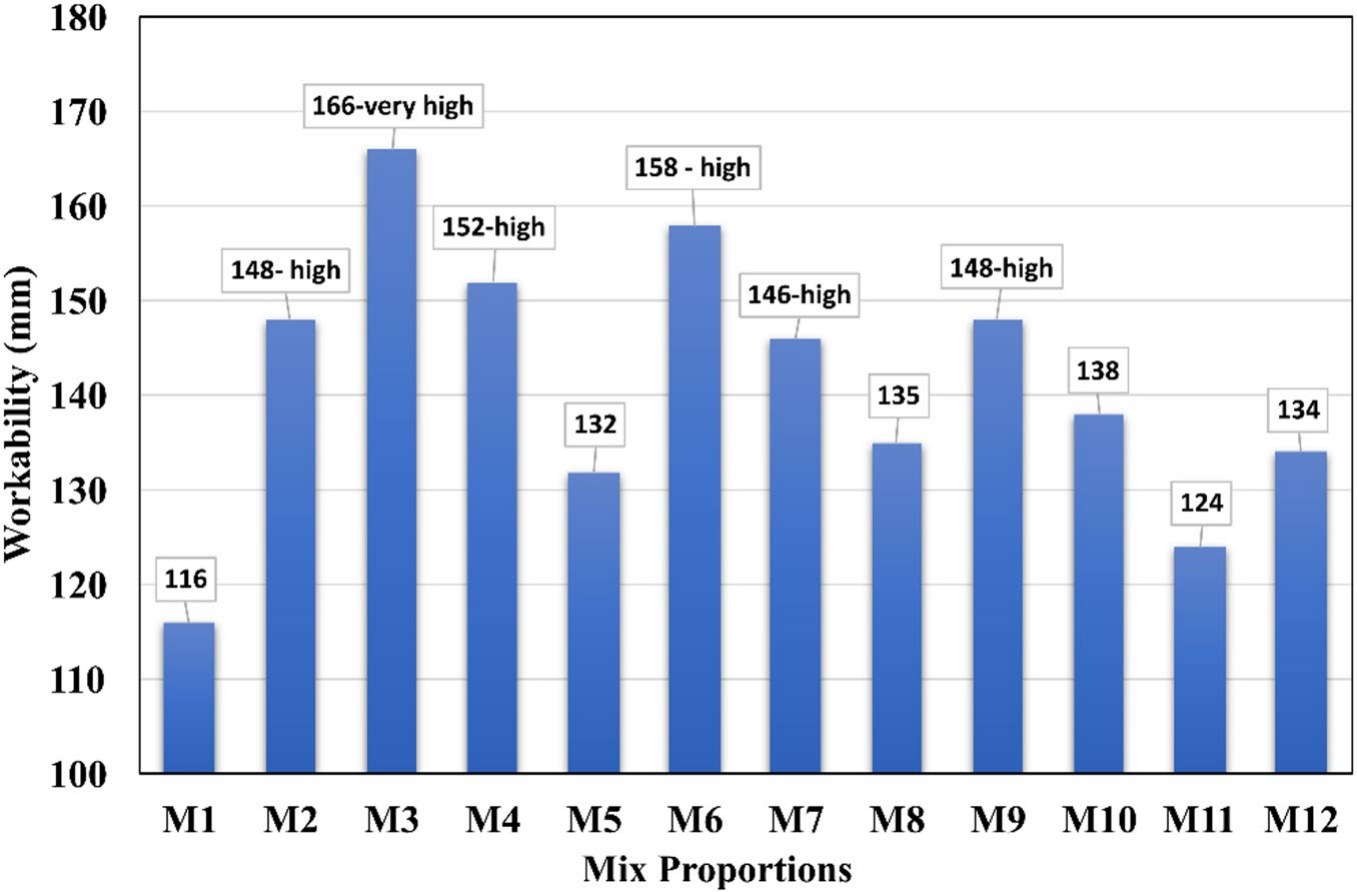
Workability test results.

In Table 4, the workability of all mixes was presented along with the quantity of elements used to prepare the mortar. The workability test result illustrated in Fig. 8, it is obvious to replace cement partially with UFFA and CLP along with BP. The control specimen shows good workability with a medium flow rate (16%), which is closely followed by M11 mix with just 7% increased deviation, which is as good as the control mortar. M8 mix with 8% deviation also with good workability with a medium flow rate of 24%. Hence, M11 and M8 mixes become most possible better combinations with respect to other mix proportions.

Workable and cohesive paste condition of the fresh mix demonstrates smooth flow, adequate plasticity, and uniform dispersion of particles without signs of segregation or excessive stiffness. Here, Mix M11 exhibited a consistency level very close to the control mix (M1), indicating that the addition of additives did not adversely affect the fresh-state behaviour.

Capillary forces arise from the thin water films between particles, generating attractive forces that influence mix cohesion and flow resistance. These forces become more significant as particle size decreases or as surface area increases. Electrostatic forces relate to the surface charge interactions among particles. Depending on the chemical composition and ion concentration in the pore solution, particles may experience repulsion or attraction, which directly affects dispersion, fluidity, and overall workability.

Due to excess amount of circular shaped fine particle size with smooth surface, mix 3 shows very high workability even with a smaller amount of water. Circular or rounded particles typically have lower surface roughness and reduced interparticle friction, allowing them to move more easily relative to one another within the paste. This reduced friction contributes to improved flowability and better workability when compared with angular or irregular particles, which tend to interlock and resist movement. Due to the ball-bearing effect, less resistance force (shear force) may be experienced by the mix M2 to M7 specimens. Therefore, high, and very high workability was noted. The mix with virtually the same proportion of UFFA and CLP (M8 to M12) shows medium or good workability with marginal variation; this is due to the cohesion of UFFA particles with CLP particles in the aid of agitated BP while reacting with water. The flowing ability of mix M9 to M11 was like that of M1 due to proper batching and mixing of UFFA, CLP and BP to form the cementitious character of cement, thereby the initial hydration reaction was rapid to form a rheopectic phase at the early stage.

**Table 4:** Workability of mortar mixes.

S. no.	Mix ID	Quantity of water (~ g)	Flow table readings (mm)	Workability	
				Flow %	Remarks
1	M1	116	116	16	Medium
2	M2	94	148	48	high
3	M3	141	166	66	Very high
4	M4	99	152	52	High
5	M5	94	132	32	High
6	M6	97	158	58	High
7	M7	105	146	46	High
8	M8	119	124	24	Medium
9	M9	112	148	48	High
10	M10	105	125	25	Medium
11	M11	107	119	19	Medium
12	M12	116	134	34	Medium

The improvement in workability with a higher proportion of lime powder is attributed to its filler effect and particle morphology. Lime powder consists of fine, smooth, and low-reactive particles, which help reduce internal friction within the fresh mix. Its fineness allows it to act as a lubricating agent, improving particle packing and enhancing the ease of flow. Additionally, lime powder has a lower water demand compared to cementitious materials, which helps retain free water in the mix, thereby contributing to increased workability. This explanation has been added to the revised discussion for better clarity.

### Compressive strength

**Fig. 9:**
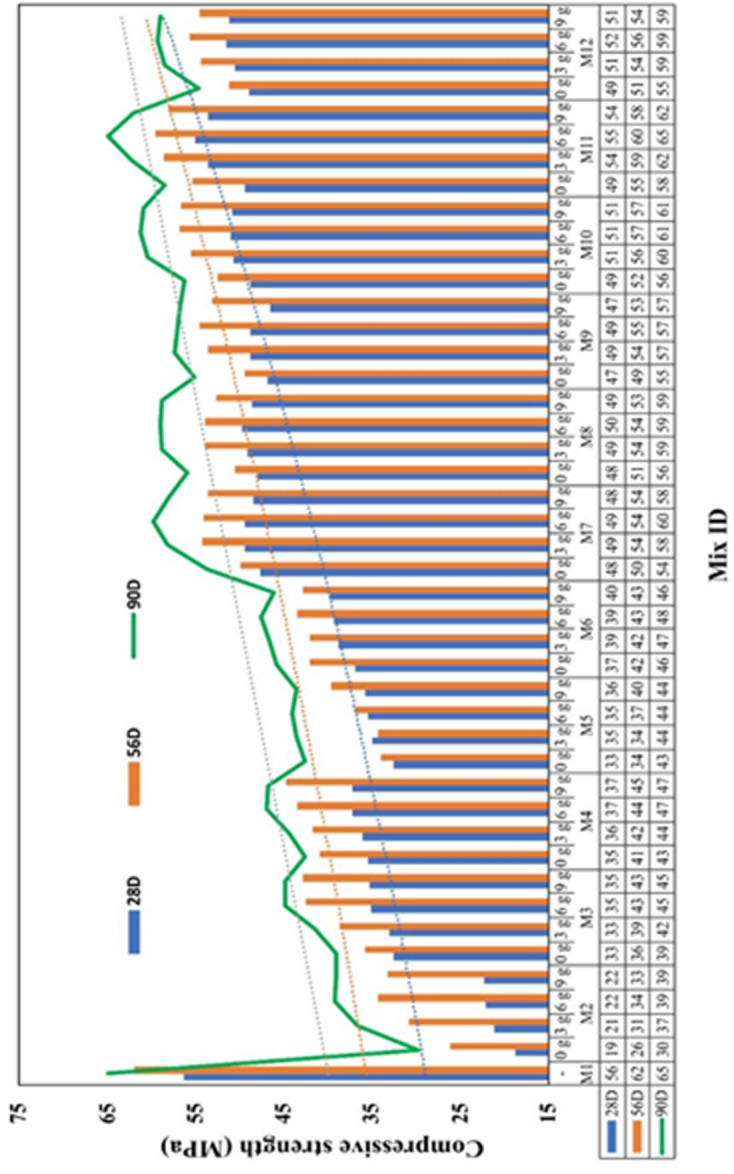
Compressive strength test result.

The test result of compression strength is plotted in Fig. 9. It is observed that the mixes with 6 g of AA outperformed the other mixes with 3 and 9 g of AA; therefore, compressive strength corresponding to 6 g was chosen for comparative evaluation. The compression strength of mix M1 with 100% of OPC provides the baseline to compare high volume fly ash mortar mix, high volume ultra-fine fly ash mortar mix and hybrid high volume ultra-fine fly ash mixes. The mix M1 shows complete hydration, forming C-S-H components that contribute to the strength development. The compressive strength of M1 for 28, 56, and 90 days is 54.25 MPa, 59.48 MPa, and 62.53 MPa, respectively, which are almost pertained with the theoretical values for the corresponding days.

The high-volume fly ash mix (M2) shows 22.12 MPa, 34.25 MPa, and 39.19 MPa as the strength for 28, 56, and 90 days testing, which are 55%, 42%, and 35% less than M1. Meanwhile, the high volume ultra-fine fly ash mix (M3) shows 35.12 MPa, 42.51 MPa, and 44.89 MPa as the strength, which are 35%, 28.5%, and 28.2% less when compared to M1. In the interim, M3 shows 19%, 13% and 6% increase in strength with respect to M2. Similar studies reported lower early-age strength but improved long-term performance with fly ash replacement^52^. This result becomes evident in the poor early strength of M2 and M3 and later strength development due to the pozzolanic character of fly ash.

The significant increase in 28-day compressive strength for high-volume fly ash mixes indicates substantial long-term formation of C-S-H, driven by the reaction between the silica in UFFA and the calcium hydroxide produced during primary hydration and from the presence of CLP^53^.

Nonetheless, high volume fly ash system shows no sign of strength development for 28th day testing due to large volume of pozzolanic nature of fly ash reacting slowly with available clinker elements. The rate of heat due to hydration process is also muffled by large volume of unreacted fly ash which further pulls down the rate of hydration, therefore, early-stage strength attainment was slow. However, high volume ultra-fine fly ash (M3) shows significant improvement in strength of 40% from high volume fly ash (M2), which exhibits the importance of reducing the particle size. As the size of fly ash changes from 12 μm to 5 μm, the strength increases approximately 40%.

In hybrid mixes, M11 alone showed 54.96 MPa, 59.86 MPa and 64.85 MPa as compressive strength for 28, 56, and 90 days of testing, which indicates identical strength values as M1. Due to improved particle packing capacity and density, which leads to denser microstructure and enhanced rate of hydration, the Blended or hybrid mix shows elevated strength pertaining to high volume fly ash. Moreover, calcium and alumina have accelerated the early hydration while mixing with ultra-fine fly ash.

In the high volume ultra-fine fly ash mortar system, since the fly ash particles are well-ground, the reactivity becomes high due to the large surface area and high packing density. This makes UFFA easily react with available calcium hydroxide and alumina provided by CLP and BP, respectively. The alkalinity of AA activates the very early stage of hydration and remains infused to drip-feed the reaction. Therefore, early strength attainment was at a moderate level, and later strength development was as likely as the control specimen.

Therefore, the mix M11 is clearly distinct from all other blended mixes, having the strength slightly more than the control specimen with 6 g of AA content. Hence, it is concluded that Mix M11 is the optimum mix regarding compressive stress.

**Table 5:** Statistical analysis for mortar samples for compression testing.

Anova: Two-factor with Replication, for compression testing samples for Fig. 6						
Source of Variation	SS	Df	MS	F	P-value	F_crit_
Sample (Mixes 2 to 12)	7125.40	10	712.54	205.10	1.8E-46	1.977
Columns (28D, 56D, 90D)	548.20	1	548.20	156.90	4.7E-19	3.986
Interaction (depending factor)	92.80	10	9.28	2.67	7.9E-03	1.977
Within (Mixes)	197.00	66	2.98	-	-	-
Total	7963.40	87	-	-	-	-

*SS – Sum of squares, df – degree of freedom, MS – Mean square, F – Statistic ratio, P – Probability value, F_crit_ – Cutoff value for F.

Table 5 shows the ANOVA results indicate that both the sample factor and curing-age factor continue to exhibit strong statistical significance, with F-values (205.10 and 156.90) far exceeding their critical limits and extremely small p-values. The interaction effect also remains significant (F = 2.67, *p* = 0.0079), showing that the influence of mixes depends on the curing period. The reduced within-group variance demonstrates that nearly all variability in the compression results is explained by the sample differences, curing durations, and their interaction. This leads to a higher R² value, indicating a stronger overall model fit.

### Flexural strength and fracture toughness

**Fig. 10:**
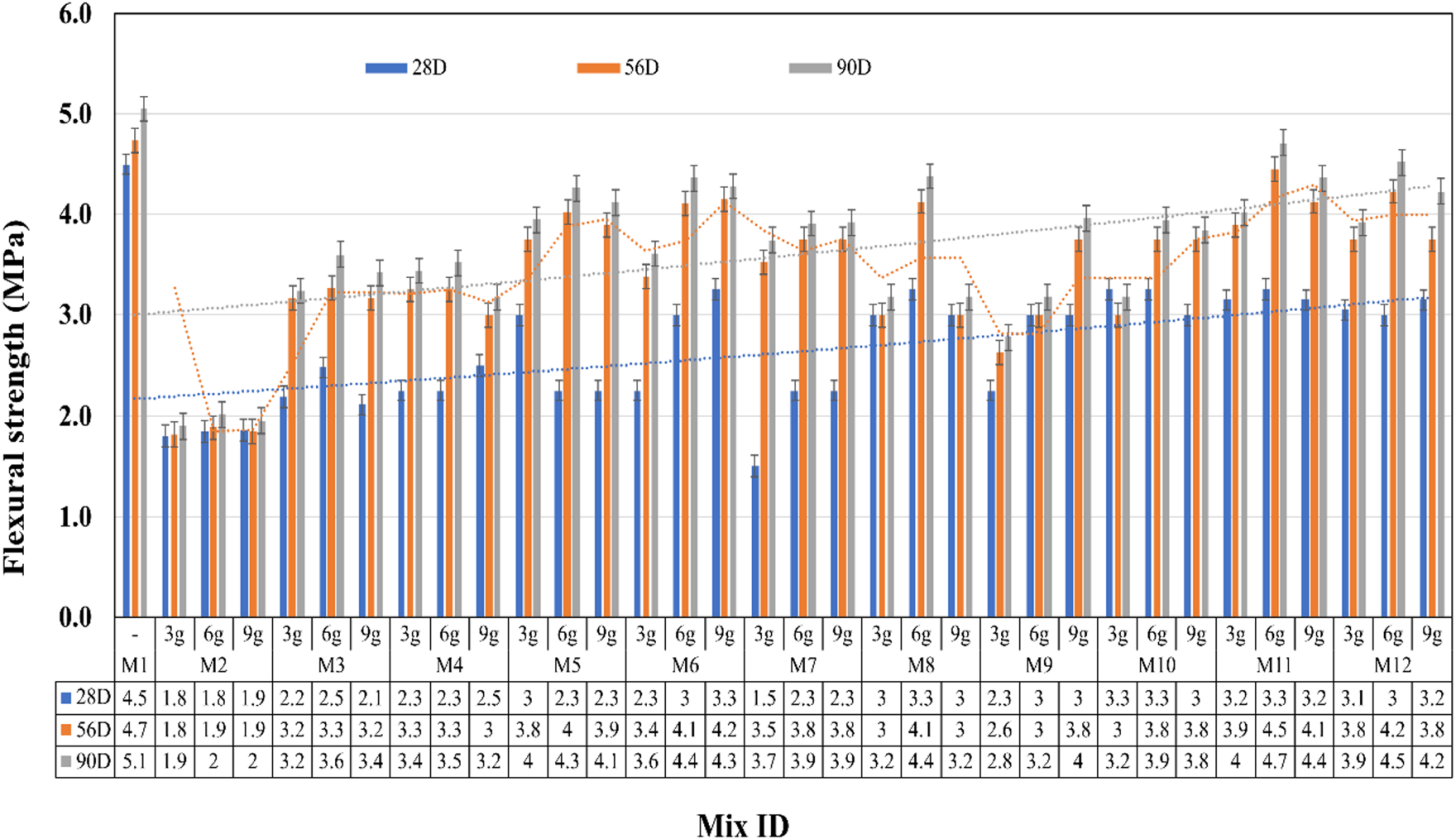
Flexural strength test results.

Figure 10; Table 5 show the results of flexural strength testing and corresponding fracture toughness for 28, 56, and 90 days of testing. As zero grams of AA does not show a significant impact on compression strength, zero grams of AA was not considered for casting prisms fsor flexural testing. The flexural strength of the control specimen was equivalent to the theoretical value meant for a 1:3 ratio for 53-grade OPC.

M1 showed 4.5 MPa, 4.73 MPa and 4.95 MPa as strength, and Mix M11 shows 3.25 MPa, 4.45 MPa and 4.93 MPa as flexural strength for 28, 56, and 90 days, respectively. Therefore, M11 showed 72% strength development at the 28th day, 98% strength development at the 56th day and 99% strength development at the 90th day. This becomes evident for later-stage strength development. Thus, M11 with 6 g of AA exhibits 28%, 2% and 1% lower stress values compared with M1 for subsequent testing days. From the result, it is obvious that M11 stands as the optimum mix in terms of flexural strength.

Equation 1 is validated when crack length to specimen width is greater than 0.2 and less than 0.8, i.e., (0.2 > a/D < 0.8). The cracks rise to grow roughly parallel to the axis of loading, and certain parts of the crack tend to form tiny branching, as shown in Fig. 4.

The cracks normally appear to propagate around unhydrated cement grains, which act as microaggregates. Pure bending cracks was occurred for all mixes, where the cracks were propagated closely from mid span soffit of the prism. The experimental testing of mortar prism illustrates the notched crack development from soffit which is in the form of ‘V’ shape from the that clearly communicates the fracture characterizes of the mortar prism as a faultless isotropic material.

**Table 6:** The fracture toughness index.

S. no.	Mix ID	AA content (g/litre)	Fracture toughness (K_Ic_) (MPa ·√m)		
			28 D	56 D	90 D
1	M1	-	1.47	1.54	1.617
2	M2	3 g	-	-	-
		6 g	-	-	-
		9 g	-	-	-
3	M3	3 g	-	-	-
		6 g	-	-	-
		9 g	-	-	-
4	M4	3 g	-	-	-
		6 g	-	-	-
		9 g	-	-	-
5	M5	3 g	-	-	-
		6 g	-	-	-
		9 g	-	-	-
6	M6	3 g	-	0.72	0.76
		6 g	0.53	0.76	0.81
		9 g	0.73	0.8	0.85
7	M7	3 g	0.32	0.76	0.81
		6 g	0.48	0.8	0.85
		9 g	0.4	0.66	0.70
8	M8	3 g	0.71	0.71	0.75
		6 g	0.73	0.8	0.85
		9 g	0.64	0.64	0.68
9	M9	3 g	0.59	0.69	0.73
		6 g	0.79	0.79	0.84
		9 g	0.88	1.1	1.17
10	M10	3 g	0.99	0.79	0.84
		6 g	0.99	0.99	1.05
		9 g	0.71	0.89	0.94
11	M11	3 g	0.99	0.99	1.05
		6 g	1.03	1.19	1.26
		9 g	0.98	0.98	1.04
12	M12	3 g	0.99	0.99	1.05
		6 g	0.99	0.98	1.04
		9 g	0.89	0.89	0.94

From Table 6, the critical value of stress intensity factor for 28, 56, and 90 days of testing is identified. The stress intensity factor of M11 shows 70% and 77% of the control specimen. This fracture mechanics symbolise that the material’s resistance to crack propagation depends upon water-cement or Cementitious ratio, age of testing, curing condition, loading rate and material type and quality. The mixes that got the ‘a/D’ ratio more than 0.8 were not considered for fracture toughness calculation.

Regarding flexure toughness, the values tabulated in Table 5 are within control limits of fracture toughness index. The Mixes M2, M3, M4, and M5 does not exhibits fracture toughness, as the ‘a/D’ ratio for the corresponding mixes are beyond the calibration limits. Stress intensity factor values indicate the amount of energy which can be stored or absorbed just before fracture of the element. In a bending member, cracks due to bending stress occur perpendicular to the tensile stress direction.

Due to the porous matrix in the initial setting stage, i.e., 28th day, the flexural bond strength was weak in M11 and much weaker in M2; the flexural strength was not adequate to compare with M1. However, the 56 and 90-day tests show significant improvement in M11 and slight improvement in M3 due to the later-stage hydration reaction, as mentioned in Sect. 5.4. The cracks were propagated along the face of the weakly bonded C-H particle with the surface of fine aggregates.

### Particle size analysis

**Fig. 11:**
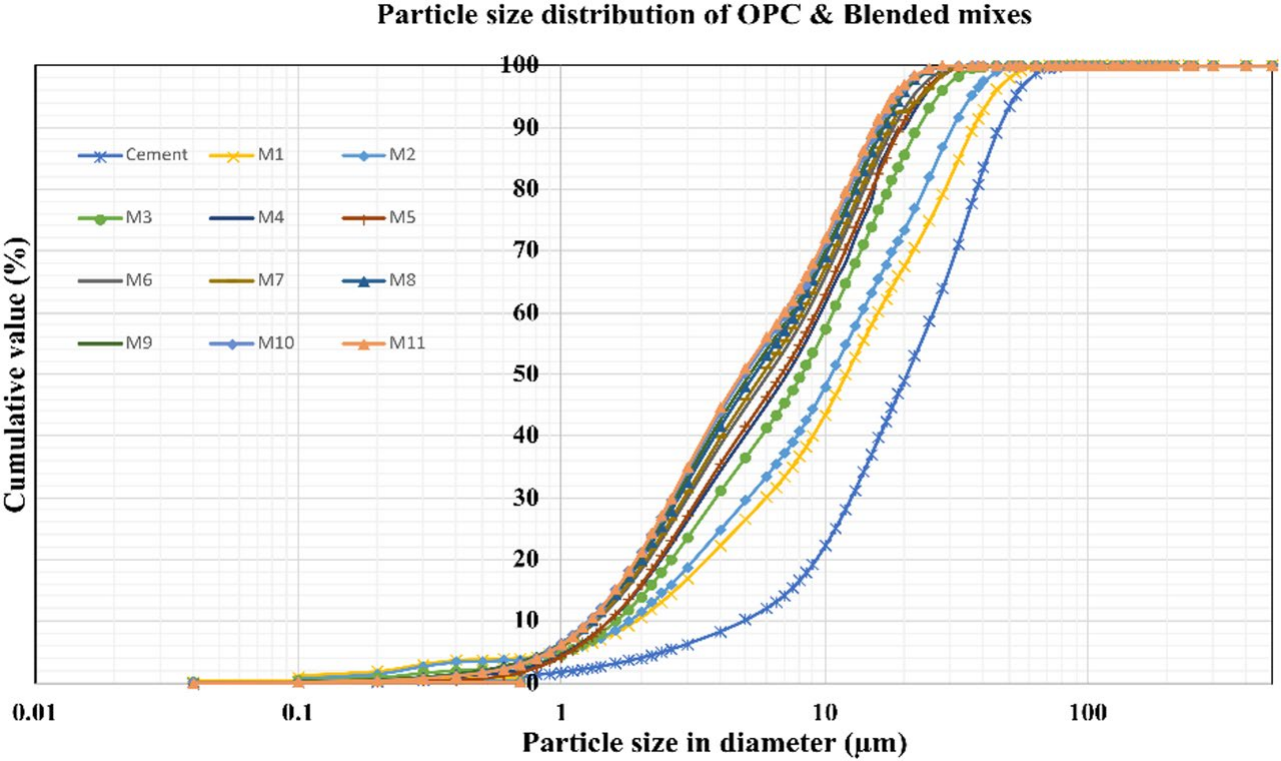
Particle size cumulative of OPC & Blended mixes.

Figure 11 infers the particle size distribution (PSD) curve for OPC and other mixes, The PSD curves indicates that OPC contains the coarsest particles, resulting in slower early hydration, while all blended mixes (M1–M11) exhibit significantly finer and more uniform particle sizes. Mixes incorporating UFFA or HVFA show the highest fineness, leading to increased surface area, enhanced reactivity, improved packing density, and a denser microstructure. Most blended mixes achieve 90–100% passing at smaller particle sizes compared to OPC, reflecting a higher proportion of fine fractions that reduce porosity and enhance durability. Although slight variations exist among the mixes due to different replacement levels, the overall trend shows that blended systems offer superior workability, faster early-age reactions, and better long-term performance than plain OPC.

### Microstructure analysis

**Fig. 12:**
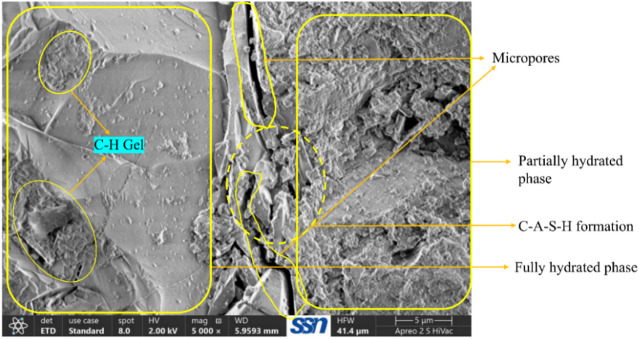
SEM image of control specimen.

**Fig. 13:**
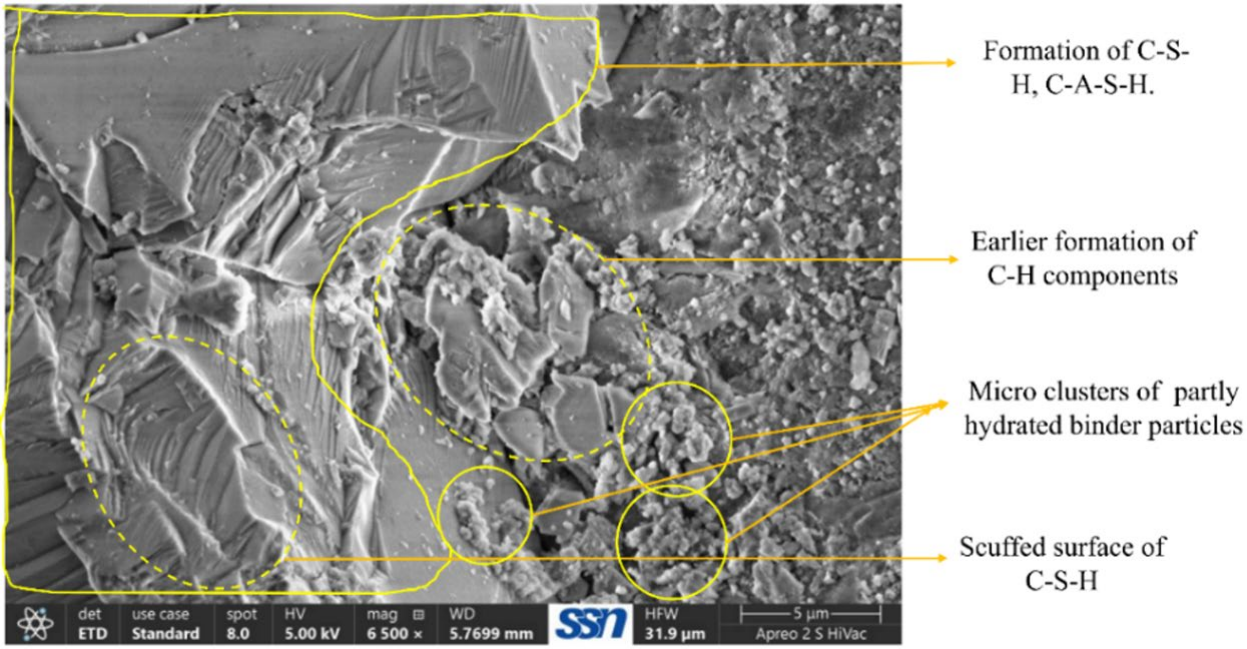
SEM image of M11 mix.

On observing the hardened state behaviour of ternary bended mixes, mix M11 with 6 g of Alkaline activator alone exhibits a comparable result with the control specimen, thereby to study the microstructure behaviour, M1 and M11 samples were adopted. Field emission scanning electron microscope (FESEM) analysis was done for the 56th day samples of the control specimen and M11 mixes, and the respective images are depicted in Figs. 12 and 13.

**Fig. 14:**
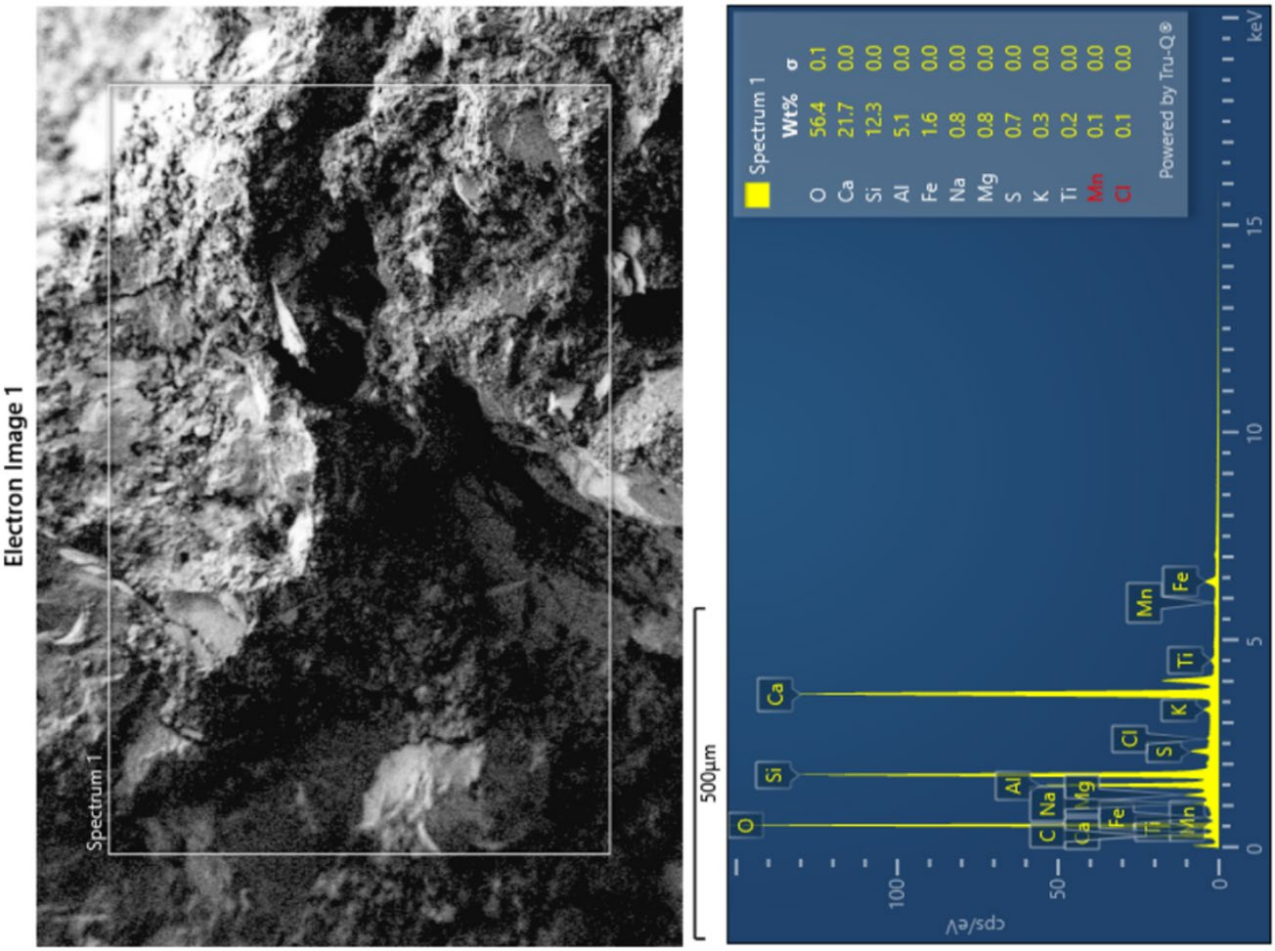
SEM-EDS analysis of sample M11.

Figure 12 shows the complete formation of ettringite, belite, and alite components. Formation of early ettringite is clearly visible in the smooth distribution pattern of reflected electrons, which includes C-H-S solidified gel and CH components. Micropores and CH gel formation are also apparent.

Meanwhile, Fig. 13 also shows delayed ettringite formation with clusters of C-H-S, which might be formed during 56 days to 90 days of curing. Figure 12 also shows the C-H-S components formed due to the ternary effect of UFFA, CLP and BP in multiple micro clusters with an aqua porous space surrounding the cluster of C-H-S components. Complete merging of UFFA, CLP and BP are occurred, as it does not show a much darker spot, which will be the result of voids or unreacted Alkaline activator. Due to the high volume of ultra-fine particles, micropores are not visible in Fig. 13 as these particles become filler materials in micropores. SEM images conclude the topographic nature and morphology of M1 and M11 mix in a comparable manner and show equivalent strength parameters.

Figure 14 illustrates combined SEM and EDS results indicate: A well-hydrated cementitious matrix dominated by C–S–H gel and calcium-rich phases. Evidence of pozzolanic reaction, supported by the presence of Si, Al, and reduced Ca compared to plain cement systems. Presence of unreacted or partially reacted particles, consistent with mixes containing fly ash or other SCMs. The microstructure suggests good long-term hydration with a mixture of dense and porous regions.

The SEM–EDS analysis confirms a hydrated cementitious system enriched with C–S–H, calcium hydroxide, and aluminosilicate phases. The morphology and elemental distribution reflect active pozzolanic reactions and ongoing development of the binding matrix, characteristic of fly ash–blended or supplementary cementitious material–based mortars.

## Conclusions

The following inferences were concluded based on the present experimental investigation on composite cementitious binder.

1. M11 and M1 show the same consistency (32%), while M2 has the lowest (20%) and M3 shows a slight increase (24%) compared to M2.
2. M2 has the longest setting time due to low CaO, whereas M11 closely matches M1 with only minor differences in initial and final setting times.
3. M11 exhibits nearly identical workability to M1, with just a 3% difference in flow rate.
4. M11 shows a 4% strength gain over M1, while M2 and M3 achieve significantly lower strengths (37% and 28% decreases).
5. M11 shows the smallest flexural strength reduction, while M2 and M3 show larger decreases. Fracture toughness of M11 approaches M1 over time, reducing the difference from 22% at 28 days to 6% at 56 days.
6. Both M1 and M11 show early C-S-H formation; M1 shows more ettringite, while M11 exhibits CH phases converting to C-S-H and better pore filling due to ultrafine particles.

The experimental results reveal the optimum proportion of ultra-fine fly ash, calcinated limestone powder and bauxite residue powder to mitigate the negative side of high-volume fly ash mortar. A promising ternary combination of 40% of UFFA, 15% of CLP and 5% of BP has been recognized as a potential replacement of cement (OPC) up to 60%.

## Recommendations for future studies

1. Examine the incorporation of other industrial waste materials such as rice husk ash, metakaolin, or ground granulated blast furnace slag in combination with UFFA, CLP, and BP.
2. Investigate the potential for achieving higher cement replacement ratios (70–80%) while maintaining performance standards.
3. Study the regional availability and variability of industrial by-products and their effects on mortar properties.
4. Investigate the performance of the sustainable mortar system in real construction applications through pilot projects.
5. Examine the bond characteristics between the sustainable mortar and reinforcement steel.

## Data Availability

Data sets generated during the current study are available from the corresponding author, Dr. S. Kandasamy ( [drskandasamy@veltech.edu.in](mailto: drskandasamy@veltech.edu.in) ) on reasonable request.
